# Development of a urine-based metabolomics approach for multi-cancer screening and tumor origin prediction

**DOI:** 10.3389/fimmu.2024.1449103

**Published:** 2024-12-13

**Authors:** Xinping Xu, Chunyan Zeng, Bei Qing, Yun He, Guodong Song, Jiaojiao Wang, Shuqi Yu, Tao Zhang, Qingyan Wei, Li Liu, He Wen, Junyuan Hu, Wei Zhang, Yan Li, Youxiang Chen, Zhenkun Xia

**Affiliations:** ^1^ Jiangxi Institute of Respiratory Disease, The First Affiliated Hospital of Nanchang University, Nanchang, China; ^2^ The First Affiliated Hospital of Nanchang University, Nanchang, China; ^3^ The Second Xiangya Hospital of Central South University, Changsha, China; ^4^ Metanotitia Inc., Shenzhen, China; ^5^ The Second Hospital of Tianjin Medical University, Tianjin, China

**Keywords:** urinary metabolomics, multi-cancer screening, tumor origin prediction, machine learning, pathway

## Abstract

**Background:**

Cancer remains a leading cause of mortality worldwide. A non-invasive screening solution was required for early diagnosis of cancer. Multi-cancer early detection (MCED) tests have been considered to address the challenge by simultaneously identifying multiple types of cancer within a single test using minimally invasive blood samples. However, a multi-cancer screening strategy utilizing urine-based metabolomics has not yet been developed.

**Methods:**

We enrolled 911 cancer patients with 548 lung cancer (LC), 177 with gastric cancer (GC), and 186 with colorectal cancer (CRC), alongside 563 individuals with non-cancerous benign diseases and 229 healthy controls (HC) and investigated the metabolic profiles of urine samples. Participants were randomly allocated to discovery and validation cohorts. The discovery cohort was used for identifying multi-cancer and tissue-specific signatures to build the cancer screening and tumor origin prediction models, while the validation cohort was employed for assessing the performance of these models.

**Results:**

We identified and annotated a total of 360 metabolites from the urine samples. Using the LASSO regression algorithm, 18 metabolites were characterized as urinary metabolic biomarkers and exhibited excellent discriminative performance between cancer patients and HC with AUC of 0.96 in the validation cohort. In comparison with the performance of traditional tumor markers CEA, the screening model performed higher sensitivity across the cancer stages, with a particularly increase in sensitivity among early-stage cancer patients. Moreover, the screening model also exhibited in high classification of cancers from non-cancerous group, comprising with HC and benign disease participants. Furthermore, two non-overlapping metabolic panels were selected to differentiate LC from Non-LC and GC from CRC with the AUC values of 0.87 and 0.83 in validation cohorts, respectively. Additionally, the model accurately predicted the origin of three lethal cancers: lung, gastric, and colorectal, with an overall accuracy of 0.75. The AUC values for LC, GC, and CRC were 0.88, 0.88, and 0.80, respectively.

**Discussion:**

Our study demonstrates the potential of urine-based metabolomics for multi-cancer early detection. The approach offers non-invasive cancer screening, promising widespread implementation in population-based programs for early detection and improved outcomes. Further validation and expansion are needed for broader clinical applicability.

## Introduction

Cancer is a leading cause of death worldwide, and it is predicted that global cancer incidence will double by 2070 compared to 2020 (1). It was estimated that in 2022, lung cancer (LC), colorectal cancer (CRC), and gastric cancer (GC) were among the five most common cancers and leading causes of cancer-related deaths in China, representing 22.0%, 10.7%, and 7.4% of new cancer cases, respectively, and contributing to 28.4%, 9.3%, and 10.1% of total cancer deaths (2). Cancer screening aids in the detection of cancer at early stage when treatment is both more effective and cost-efficient. To date, the clinical blood tumor biomarkers are limited, including alpha-fetoprotein (AFP), cancer antigen 19-9 (CA19-9), and carcinoembryonic antigen (CEA), all of which demonstrate low sensitivity. Besides, most of the current screening tests are for single cancer, which may lead to elevated false positive rates when used consecutively (3). Therefore, there is an urgent need for non-invasive screening solution for pan-cancer early diagnosis.

Multi-cancer early detection (MCED) tests have the potential to address these challenges by identifying multiple types of cancer simultaneously within a single test, with numerous novel technologies being investigated (4). For example, a targeted cfDNA methylation-based assay called Galleri test was applied to diagnose 50 different cancers (5). Moreover, the CancerSEEK test integrates genetic mutation signals and protein markers to detect eight types of cancer (6). However, these methods require complex sample processing and high depth coverage of sequencing, which are time-consuming and costly, hindering the population wide screening (7). Previous studies have shown the potential application of metabolomics in diagnosing certain cancers, as cancer is acknowledged as a metabolic disorder (8). Additionally, metabolomics technologies exhibit high reliability and reproducibility, making them promising tools for identifying potential biomarkers in tissues, blood, and urine samples (9). Compared to other biological fluids (tissue and blood), urine has the advantages of being inexpensive, easy to handle, and available in large amounts, without requiring invasive treatments for collection, which could potentially broaden its application (10). Besides, it contains a variety of inorganic salts, organic compounds, and diverse exfoliated cell types, providing rich metabolic information reflecting the systemic metabolic status (11, 12). Moreover, urine as a carrier for blood wastes, contains various metabolic products from pathways (13). Hence, the application of urine-based metabolomics in cancer screening is poised to become a powerful tool for clinical cancer diagnosis. Currently, urinary metabolomics has been applied to identify potential markers for the diagnosis of various cancers. For instance, meta-analysis characterized eleven relevant urinary metabolites for colorectal cancer diagnosis (14). In addition, Mathé et al. (15) and Chan et al. (16) revealed specific urinary metabolic biomarkers of diagnosing lung cancer and gastric cancer, respectively. This demonstrated the clinical potential of urinary metabolomics for cancer diagnosis. To the best of our knowledge, however, a comprehensive multi-cancer screening strategy utilizing urine-based metabolomics has not been developed yet.

Herein, the aim of this study is to develop a urinary metabolomics-based approach for multi-cancer diagnosis by identifying cancer-specific urinary biomarkers, enabling the construction of screening models that can distinguish between cancerous and non-cancerous individuals and accurately predict the origin of three lethal cancers (including LC, CRC, and GC). The urinary metabolomics holds promise of becoming a universally applicable, straightforward, and cost-effective means of early detection for these three cancers across large populations.

## Methods

### Study population

A case-control study was conducted between September 2018 and January 2020 at three independent centers, including the First Affiliated Hospital of Nanchang University, the Second Xiangya Hospital of Central South University, and the Second Hospital of Tianjin Medical University. Participants included individuals with LC, GC, CRC, non-cancer benign diseases (NCD) of the lung, stomach, and colorectum, as well as healthy controls (HC). The study was approved by the local Institutional Review Board, adhering to the guidelines of the International Conference on Harmonization for Good Clinical Practice and the Declaration of Helsinki, with formal consent obtained from the participants. All participants were screened as described previously (17). Morning fasting urine samples were collected, centrifuged, and processed using established methods (17).

### Sample preparation and metabolites detection

The extraction buffer, consisting of methanol, methyl tert-butyl ether, water, and a mixture of chemical standards (gibberellic acid A3: 0.45 µg/mL, ^13^C sorbitol: 1 µg/mL, and PE (17:0/17:0): 1 µg/mL), was combined with the samples and sonicated for 15 minutes at 4°C. Following this, 350 µL of methanol/water (v/v, 1:3) was added to facilitate phase separation. The resulting upper lipophilic phase and hydrophilic phase were collected separately after high-speed centrifugation (18000 g, 5 minutes at 4°C, Centrifuge 5430R, Eppendorf, Germany). All aliquots were dried and stored at -80°C for further analysis. The polar fraction was derivatized according to Lisec et al. (18). Metabolite analysis was performed using gas chromatography and UPLC-MS (Waters ACQUITY ultra performance liquid chromatography (UPLC) system coupled to Thermo-Fisher Q-Exactive mass spectrometers with an electrospray ionization (ESI) source). The resuspended samples were injected into a Waters ACQUITY FTN autosampler set to a temperature of 10°C. Data were acquired in both positive and negative modes, with parameters as described previously (17).

### Data processing

The raw data (.raw) were initially processed using Metanotitia Inc.’s in-house developed software PAppLine™. This process involved peak picking, baseline correction, alignment, removal of isotopic peaks, annotation, and baseline noise removal. Metabolic features detected in less than 80% of the subject samples were discarded to mitigate the impact of noise and outliers in the data. Missing values were addressed separately for the subject samples and the quality control samples (QC_Nist_), using the same strategy batch-by-batch. The imputation method varied depending on the missing rate and average intensity of each feature within the batch. For features with a low missing rate and high average intensity, the MICE forest algorithm was applied. For those with a high missing rate or low average intensity, the half-minimum method was used for imputation. Meanwhile, features with high missing rate or low averaging intensity were imputed by the half minimum strategy. The MSTUS (total useful MS signals) method is used to normalize mass spectrometry data to eliminate dilution effects present in urine samples (19). Subsequently, normalization was conducted utilizing the QC-based deep learning method, NormAE algorithm. Finally, a calibration procedure, including logarithm transformation and scaling, was employed.

### Feature selection and model construction

Feature selection was independently conducted for each platform within the discovery cohort using the least absolute shrinkage and selection operator (LASSO) algorithm. The LASSO method regularizes parameters of a linear regression model by reducing some coefficients to zero, allowing the selection of features with nonzero coefficients (20). To enhance its stability, we applied an ensemble learning approach, constructing and amalgamating multiple selection systems with a recurring random data-splitting strategy. Subsequently, selected features from the three distinct MS-based platforms were integrated for addressing the forthcoming classification problem. For the training model, a five-fold cross-validation procedure repeated five times was applied to each classifier. The discovery set was divided into two subsets at a ratio of 4:1 to compute the training error on the resulting 20% dataset. Through five repetitions of this process, the average training error was calculated and employed for training hyper-parameters. The model comprises the Balanced Support Vector Machine (SVM) algorithm, a fundamental classification algorithm. These techniques are adept at handling high-dimensional mass spectrometry-based data, particularly in situations with limited sample sizes. To tackle multi-class classification, we used the One vs. Rest approach for these three distinct cancer types and employed ensemble learning techniques like bagging and stacking to enhance the metabolic panel (MP) model. For the test model, parameters were determined during training, and the validation set was used to compute validation error, including confusion matrix, ROC curves, and AUC, for all corresponding classifiers.

### Statistical analysis

The data’s statistical significance was assessed at a 95% confidence level (*p* < 0.05) using binomial distribution. Data processing and machine learning were conducted in Python (version 3.10.12), leveraging the Numpy (version 1.23.5) and Pandas (version 1.5.3) libraries. Imputation was carried out with Miceforest (version 5.6.2), while feature selection and machine learning employed Scikit-learn (version 1.2.2) and Scipy (version 1.10.1). Matplotlib (version 3.8.2) was employed for generating the confusion matrix to summarize classification results and plot ROC curves. The confusion matrix for multiple classification (among three cancers: GC, CRC, and LC) was generated using Scikit-learn metrics. The heatmap was drawn using R (version 4.2.0). To assess the accuracy, sensitivity, and specificity of the different groups, ROC curves were applied for various scenarios (discovery set, validation set). ROC curves and AUC values were performed by Scikit-learn.

## Results

### Characteristics of participants

**Figure 1:**
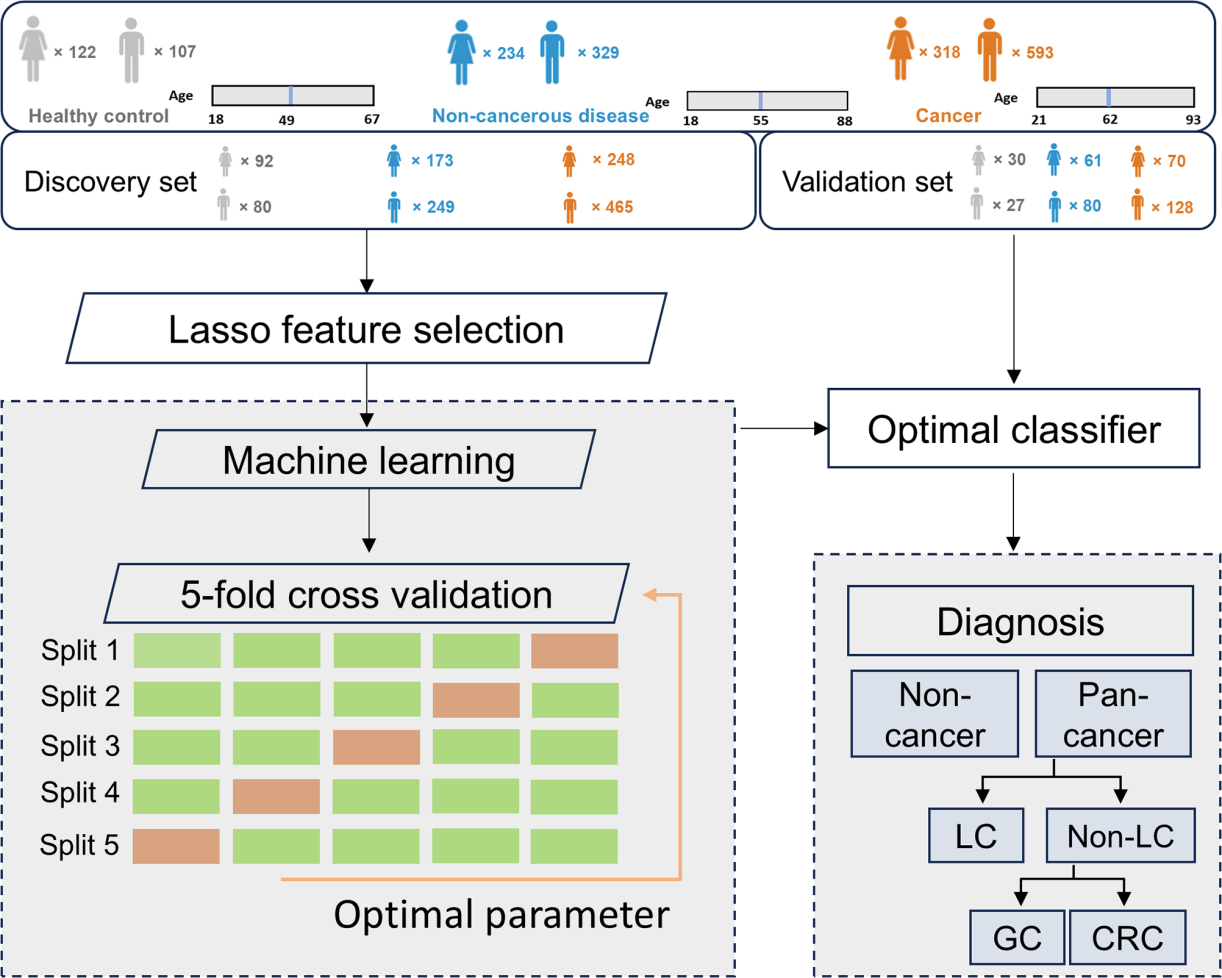
The workflow diagram of study design.

This study recruited participants with three common types of cancer: LC (n = 548), GC (n = 177), and CRC (n = 186), along with 563 patients with non-cancerous benign diseases (NCD) and 229 healthy controls (HC). All cancer patients were diagnosed by histopathology and/or imaging techniques, depending on cancer types, and had not received any prior treatment at the time of urine collection. The cancer, non-cancer disease, and HC groups were randomly assigned to the discovery and validation cohorts (**Table 1**). The discovery cohort was utilized for profiling multi-cancer- and tissue-specific signatures and constructing machine learning algorithms, whereas the validation cohort was exclusively employed for evaluating the performance of the machine learning models (**Figure 1**).

**Table 1:** Baseline characteristics of the participants.

Variable		Discovery Cohort (N=1307)			*p* value	Validation Cohort (N=396)			*p* value
		Cancer (N = 713)	NCD (N = 422)	HC (N = 172)		Cancer (N = 198)	NCD (N = 141)	HC (N = 57)	
Age, median (range)		62 (21-93)	56 (18-87)	47 (18-67)	0.0006	63 (27-82)	53 (19-88)	50 (23-66)	0.0016
Gender, N (%)	Female	248 (34.8%)	173 (41%)	92 (53.5%)		70 (35.4%)	61 (43.3%)	30 (52.6%)	
	Male	465 (65.2%)	249 (59%)	80 (46.5%)	0.4793	128 (64.6%)	80 (56.7%)	27 (47.4%)	0.4784
Stage, N (%)	I	214 (30%)				60 (30.3%)			
	II	80 (11%)				19 (9.6%)			
	III	123 (17%)				36 (18.2%)			
	IV	107 (15%)				31 (15.7%)			
	Non-metastasis with unknown staging information	189 (26.5%)				52 (26.3%)			

NCD, non-cancerous disease; HC, healthy control.

The discovery cohort included 713 cancer patients (431 LC, 142 GC, and 140 CRC), 422 NCD patients, and 172 healthy controls. The gender ratios were comparable between the cancer, NCD, and HC groups (*p* > 0.05), while the cancer patients were found to be the oldest than the other two groups with a median age of 62 (*p* < 0.05) (**Table 1**). To better establish an early cancer screening model, we enrolled numerous early-stage cancer patients, with 30% of them diagnosed at stage I. The validation cohort comprised of 198 cancer patients, including 117 LC, 35 GC, and 46 CRC, 141 NCD patients, and 57 HC. Consistent with the discovery cohort, the gender distribution was comparable between the cancer, NCD, and HC groups. Furthermore, the cancer group had a higher median age (63 years) compared to the other two groups (53 and 50 years) (*p* < 0.05). The distribution of cancer patients across each stage closely resembled that of the discovery cohort (**Table 1**).

### Establishment of metabolites-based signature panel for pan-cancer detection

**Figure 2:**
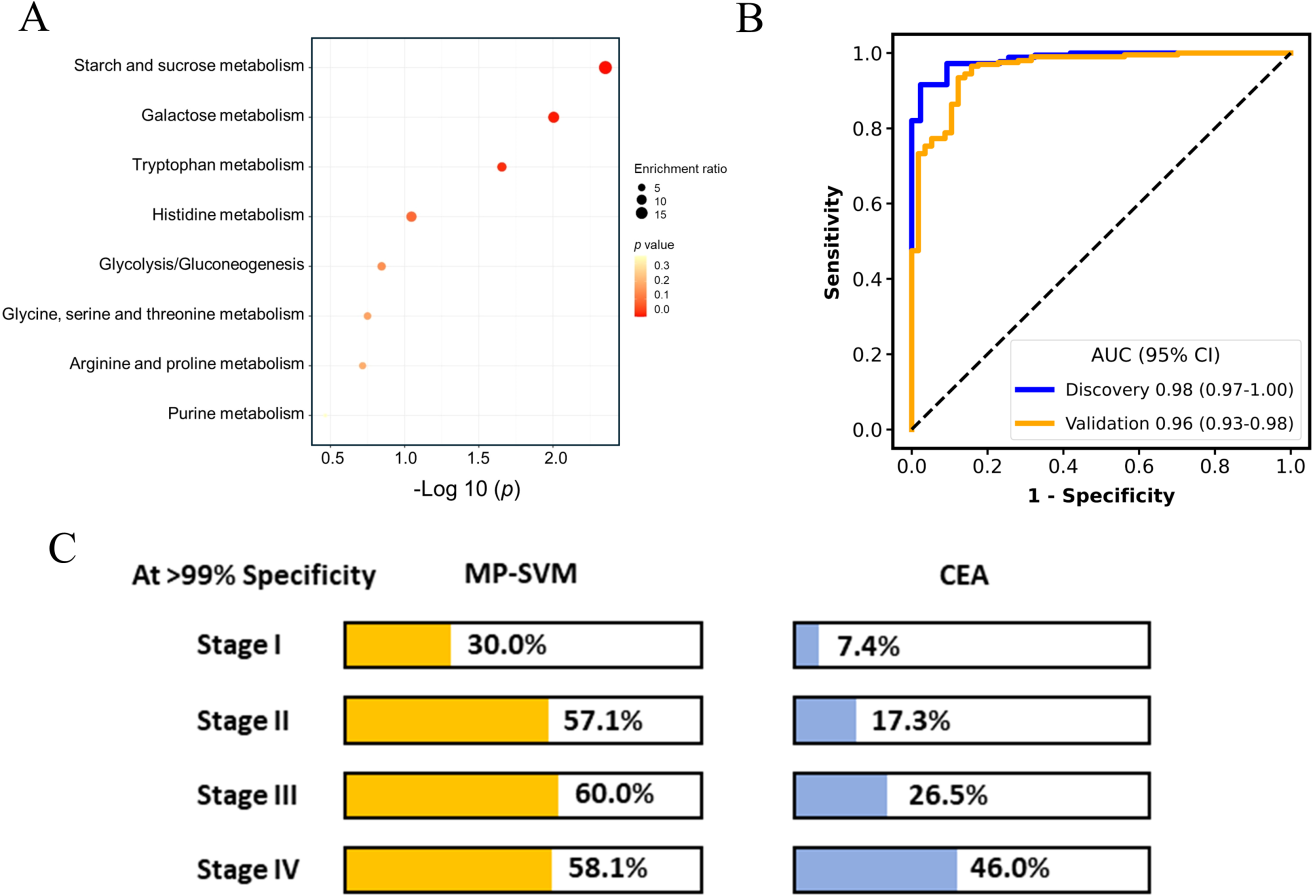
The performance of metabolic panel-based model for discriminating cancer patients from healthy controls. Kyoto Encyclopedia of Genes and Genomes (KEGG) metabolic pathways enriched by least absolute shrinkage and selection operator (LASSO) selected features **(A)**. The receiver operating characteristic (ROC) curve for the diagnosis of cancer patients vs. healthy controls **(B)**. Detection rates of metabolic panel-based model and carcinoembryonic antigen (CEA) at > 99% specificity in the validation cohort **(C)**.

After peak alignment and removal of missing values, 125, 13, and 222 metabolites were identified and annotated on the polar, lipid, and GC platforms, respectively, including fatty acids, carbohydrates, amino acid and derivatives, nucleotides and derivatives, acetyl-CoA, diacylglycerol and others. The metabolic reprogramming of cancer cells has extensively been examined in prior studies, shedding light on the alterations in patients’ metabolism (8). The 18 metabolites for the discrimination of pan-cancer and non-cancer group have been selected using the LASSO regression algorithm. KEGG pathway enrichment analysis on the metabolites panel uncovered a variety of disrupted metabolic pathways including the starch and sucrose, galactose, and tryptophan metabolism (*p* < 0.05, **Figure 2A**), which has been well characterized in cancer patients in previous research (21, 22). Subsequently, an SVM algorithm was employed to establish the screening model based on metabolic panel selected from the discovery dataset (named as MP-SVM), and the performance was further evaluated in the validation dataset. Impressively, the MP-SVM model achieved an area under the receiver operating characteristic (AUC) of 0.98 (95% confidence interval (CI): 0.97-1.00) and 0.96 (95% CI: 0.93-0.98) for distinguishing cancer group from HC in the discovery and validation datasets, respectively (**Figure 2B**). To evaluate the screening advancement of the MP-SVM model, we compared its sensitivity with that of the established clinical tumor biomarker CEA in the validation cohort. The MP-SVM model outperformed the traditional tumor marker CEA. The detection rate of MP-SVM model in the pan-cancer group was higher than that of CEA with specificity > 99% in stages I, II, III, and IV (**Figure 2C**).

**Figure 3:**
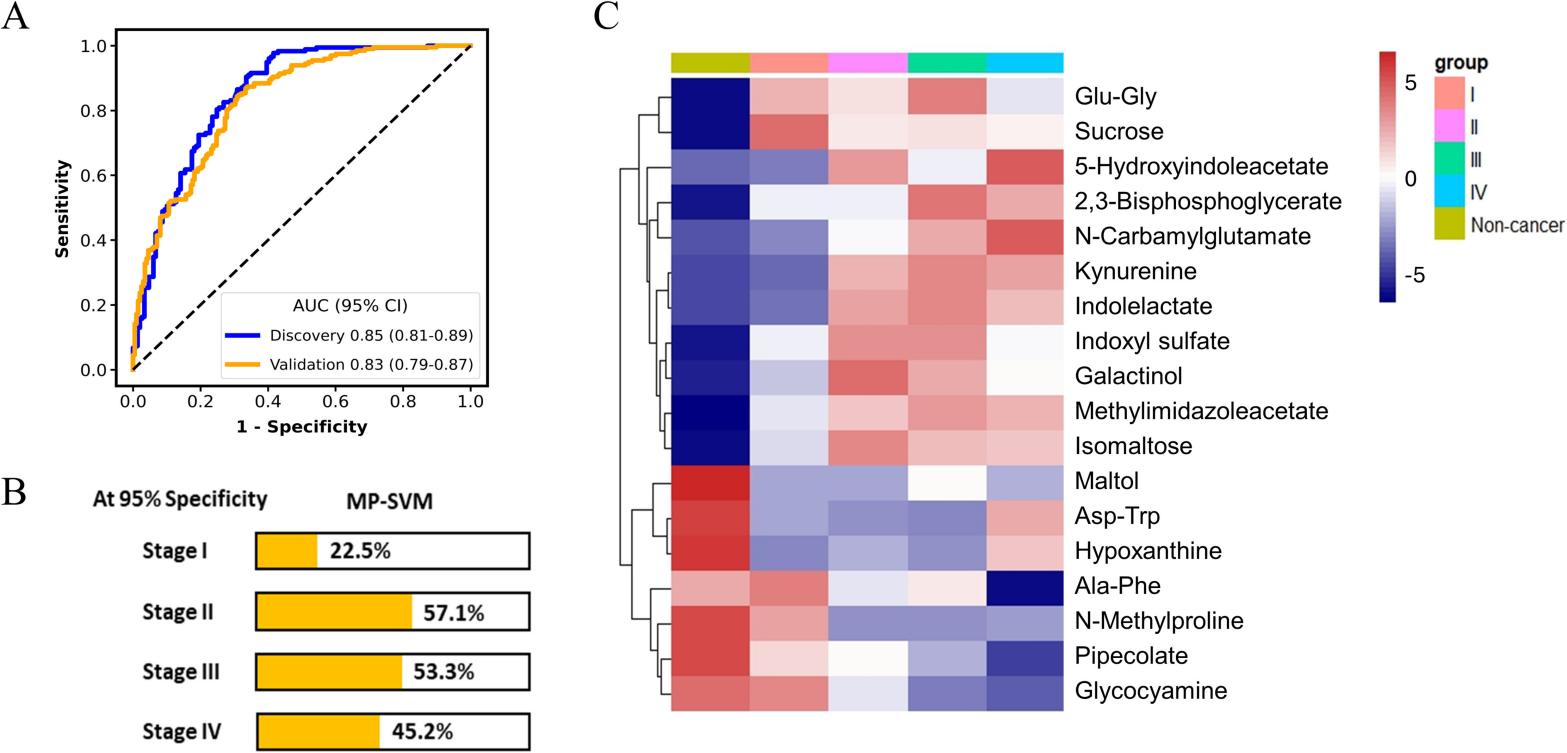
The performance of metabolic panel-based model for discriminating cancer patients from non-cancer group. The receiver operating characteristic (ROC) curve for the diagnosis of cancer patients **(A)**. Detection rates of metabolic panel-based model at 95% specificity in the validation cohort **(B)**. Heatmap of 18 least absolute shrinkage and selection operator (LASSO)-selected metabolites in non-cancer group and the cancer group at different stages **(C)**.

Moreover, the panel exhibited an AUC of 0.83 for discerning cancer group from the non-cancerous group including the HC and NCD patients in the validation cohort (**Figure 3A**). The detection rates of the MP-SVM model in the pan-cancer group with a specificity of 95% for stages I, II, III, and IV were 22.5%, 57.1%, 53.3%, and 45.2%, respectively (**Figure 3B**). The heatmap was applied to delineate the metabolite trajectories (LASSO-selected metabolites) in stage I, II, III, and IV (**Figure 3C**). The distinct variations of these metabolites were observed across the four stages of cancer, reflecting intricate shifts in metabolic dynamics throughout cancer progression. More specifically, the changes in most metabolite levels in stage I were not pronounced (e.g., 5-Hydroxyindoleacetate, N-Carbamylglutamate, kynurenine, and indolelactate). And the levels of certain metabolites in stages II and III significantly increased (e.g., kynurenine, indolelactate, indoxyl sulfate, galactinol, methylimidazoleacetate, and isomaltose). The 5-Hydroxyindoleacetate and N-Carbamylglutamate in stage IV showed the most significant increase, while pipecolate and glycocyamine exhibited the most notable decrease. Next, we assessed the potential confounding effect of age on our model by examining the correlation between the model prediction scores and the participants’ ages. The results exhibited no significant correlation (R = 0.05, *P* = 0.32), indicating that age differences are unlikely to impact the accuracy of our model (**Supplementary Figure S1**).

### Establishment of cancer classification model for tumor origin prediction

**Figure 4:**
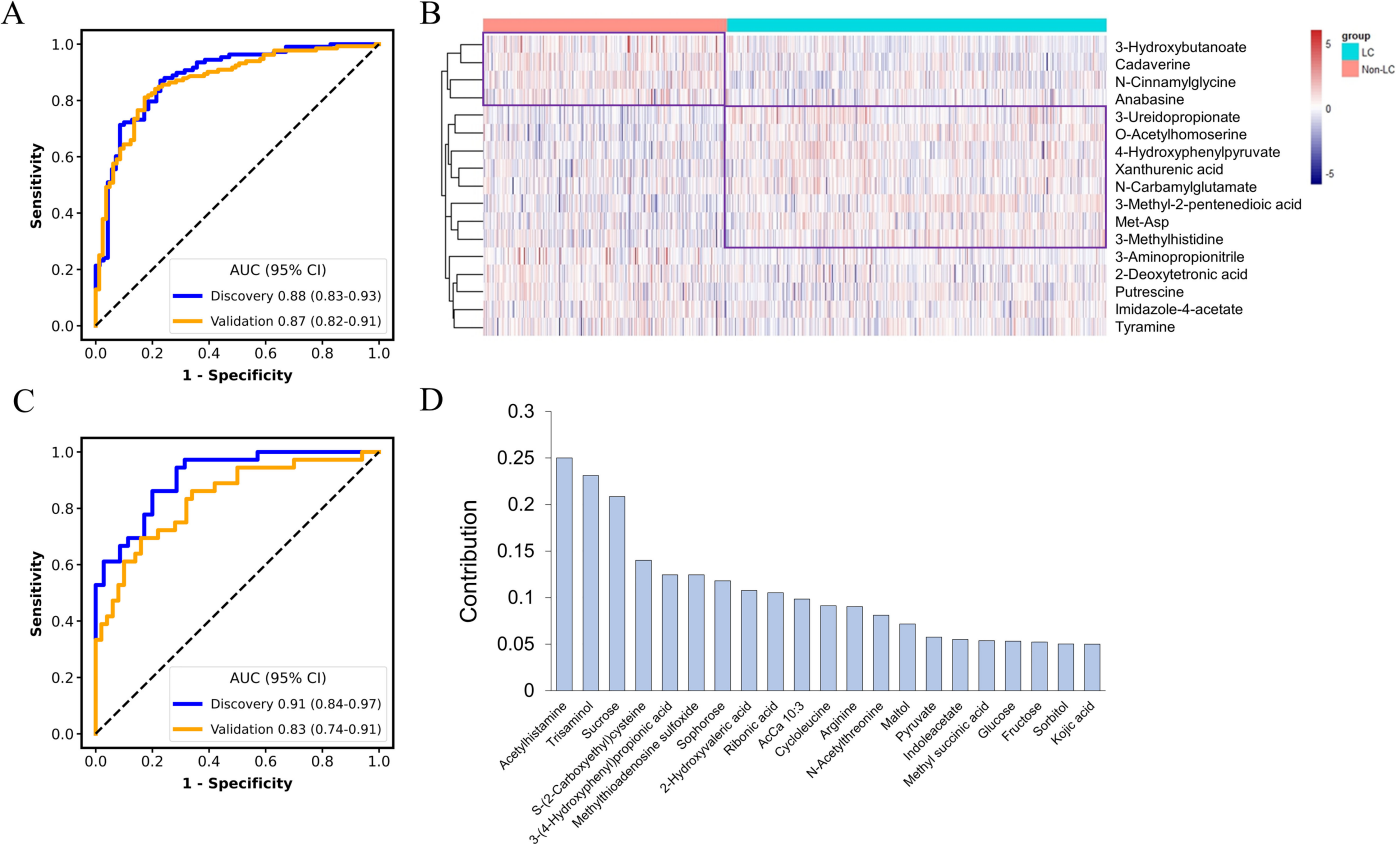
Classification of cancers by metabolic panel-based model. The receiver operating characteristic (ROC) curve for the discrimination of lung cancer (LC) patients from non-lung cancer patients **(A)**. Heatmap of the least absolute shrinkage and selection operator (LASSO)-selected metabolites between LC and non-LC patients **(B)**. The ROC curve for the discrimination of gastric cancer patients (GC) from colorectal cancer patients (CRC) **(C)**. Contribution of the selected metabolites to the discrimination model for GC vs. CRC **(D)**.

The accurate prediction of the tumor origin is essential in early cancer detection, as it guides subsequent diagnostic procedures and treatment decisions (23). Given that both GC and CRC originate in the digestive tract, they have been reported to exhibit certain shared metabolic characteristics (24). Thus, in this study, the feature selection process for the cancer classification model consisted of two stages: distinguishing between LC and non-LC samples (GC and CRC), and then differentiating between GC and CRC samples. Seventeen metabolites were selected by LASSO algorithm to differentiate LC from Non-LC, achieving AUC values of 0.88 (95% CI: 0.83-0.93) and 0.87 (95% CI: 0.82-0.91) in the discovery and validation datasets, respectively (**Figure 4A**). Among these metabolites, metabolites such as 3-Hydroxybutanoate, cadaverine, N-Cinnamylglycine, and anabasine exhibited upregulation in the non-LC group, whereas 3-Ureidopropionate, O-Acetylhomoserine, 4-Hydroxyphenylpyruvate, xanthurenic acid, N-Carbamylglutamate, 3-Methyl-2-pentenedioic acid, methionyl-aspartate (Met-Asp), and 3-Methylhistidine were found to elevate in the LC group (**Figure 4B**). These 17 metabolites were further mapped to the KEGG metabolic pathways for pathway analysis. Histidine metabolism, glutathione metabolism, tyrosine metabolism, and phenylalanine, tyrosine, and tryptophan biosynthesis were the most significantly changed pathway when comparing LC and non-LC groups (*p* < 0.05) (**Supplementary Figure S2A**).

**Figure 5:**
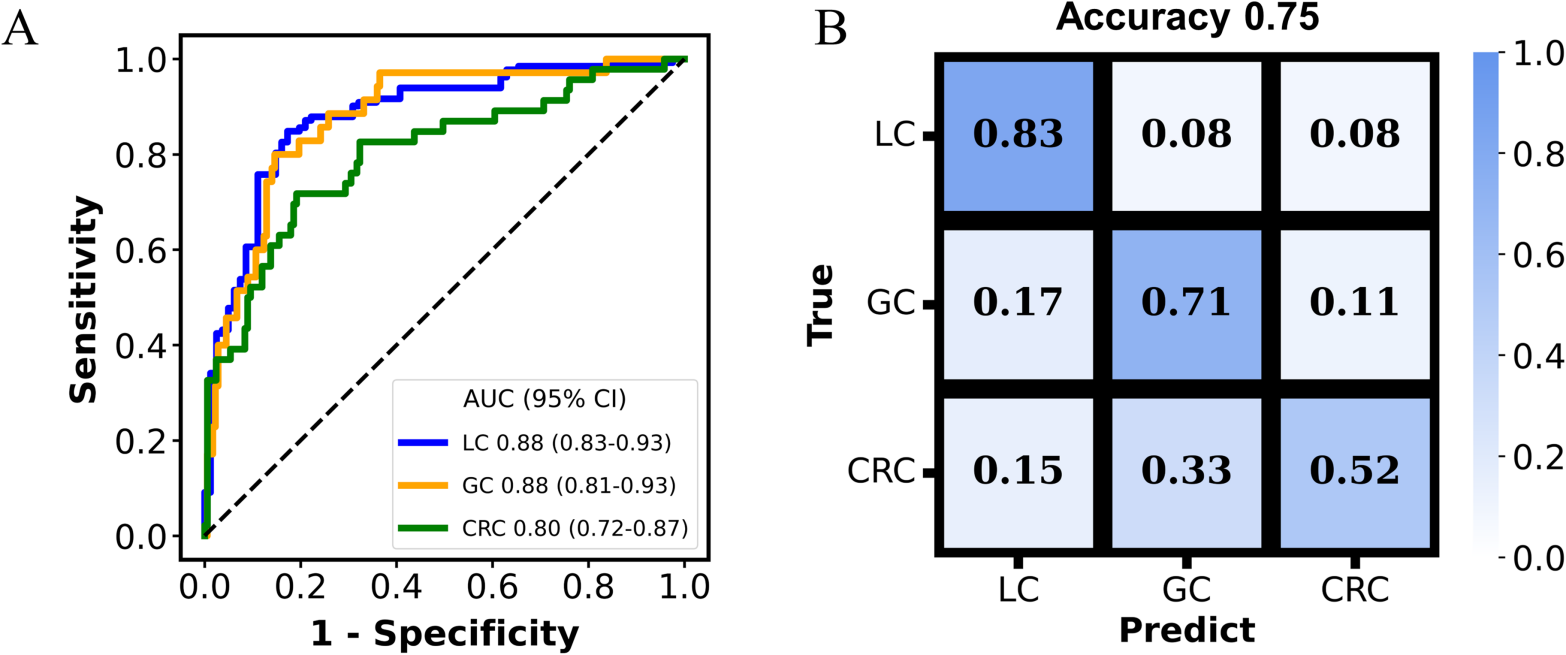
The performance of the multi-cancer classification model. The receiver operating characteristic (ROC) curves evaluating the model in discriminating tumor origins in the validation cohort **(A)**. Confusion matrix summarizing the cancer classification results in the validation cohort **(B)**.

An additional set of 21 metabolites were selected for classifying GC and CRC, without overlapping with features selected for the LC and non-LC classification. The AUC for the MP-SVM model in the discovery and validation datasets were 0.91 (95% CI: 0.84-0.97) and 0.83 (95% CI: 0.74-0.91), respectively (**Figure 4C**), with acetylhistamine, trisaminol, and sucrose being the three most significant contributing metabolites (**Figure 4D**). The 21 metabolites were enriched in 17 metabolic pathways, with 5 pathways (galactose metabolism, carbohydrate metabolism, fructose and mannose metabolism, neomycin, kanamycin, and gentamicin biosynthesis, arginine, and proline metabolism) exhibiting significant differences (*p* < 0.05) (**Supplementary Figure S2B**). The two metabolites’ panels were pooled together, forming the basis for constructing the tumor origin classification model. In the validation cohort, the model yielded an accuracy of 0.75, with AUC of 0.88 (95% CI: 0.83-0.93) for LC, 0.88 (95% CI: 0.81-0.93) for GC, and 0.80 (95% CI: 0.72-0.87) for CRC, respectively (**Figure 5A**). Among the three cancer types, LC showed the highest detection rate (0.83), while CRC exhibited the low detection rate (0.52) (**Figure 5B**).

## Discussion

In this study, we developed a novel urine metabolites-based model and revealed urinary metabolic biomarkers exhibiting high discriminative capacity for multi-cancer early detection. Despite the wide range of genetic changes observed in various types of cancer, there is a possibility that cancer development could impose similar requirements on cellular metabolism, regardless of its tissue of origin (25). Besides, subtle distinctions exist among cancers originating from different sites, which can be also identified through metabolomics. The common patterns of these biomarkers selected by LASSO across various cancers in our study, predominantly corresponded to dysregulated pathways in cancer, as reported in previous research, encompassing carbohydrate metabolism and amino acid metabolism (22, 25). Specifically, it exhibited outstanding performance by adeptly discerning cancers from healthy subjects, comparable to the performance of the model built by Zhang et al. (26), which incorporated blood-derived metabolites. Differently, our model utilized less invasive urine samples. Furthermore, besides effectively discerning between healthy individuals and cancer patients, we also exhibited robust discriminative capability between cancer patients and individuals with benign conditions, as these systemic factors have been documented to influence the metabolic profile (27).

During the early stages of tumor development, there is a demand for nutrient uptake and biosynthesis, leading to the metabolic reprogramming (28). Remarkably, in our investigation, we noted a decline in the model’s sensitivity for detecting cancer at stage IV compared to the earlier stages, such as stages II and III in comparison with the non-cancerous group. We hypothesize that the irregular variations in selected metabolites across various stages of cancer progression could be the underlying factor, corroborated by prior research indicating the specific metabolic changes in tumor progression (28). For instance, MYC, a proto-oncogene, promotes the conversion of pyruvate to alanine, whereas in advanced malignant lesions, stimulates the transformation of pyruvate into lactate (29). Investigation of the metabolic evolution from preneoplasia to lung adenocarcinoma, researchers observed a gradual alteration in metabolic pathways, suggesting that cancer progression may not uniformly change metabolic patterns (30). In another study, metabolic profiles of patients with chronic obstructive pulmonary disease (COPD) were compared with those of lung cancer patients across various stages. The findings revealed notable differences in certain metabolite levels between early-stage lung cancer and COPD patients, while metabolite levels in late-stage lung cancer patients closely resembled those of COPD patients. This suggests that the serum metabolome may reflect lung dysfunction and systemic changes in advanced stages, which are absent in early stages (31). This finding may suggest the advantages of using metabolomics for early diagnosis of cancer, however, the specific mechanism needs to be further investigated.

In a cancer-screening scenario, a high specificity was uniformly predefined to diminish false positivity and thereby prevent overdiagnosis and anxiety. However, such specificity may compromise sensitivity to some extent, potentially undermining the benefits of multi-cancer early detection. For individuals with high-risk factors such as ulcerative colitis or adenomatous polyps for CRC (23), emphysema, chronic bronchitis, pneumonia, and tuberculosis for lung cancer (32), atrophic gastritis, and *H. pylori* infection for GC (33), a higher sensitivity is preferable, even with a slight compromise in specificity. In our study, the higher specificity of the model (> 99%) may be applicable for low-risk populations, which showcased higher diagnostic sensitivity compared to conventional blood tumor marker-CEA, indicating its potential suitability for routine health check-up. The slightly lower specificity (95%) for discriminating cancer patients from non-cancerous groups (including HC and NCD) might be more suitable for high-risk populations to reduce the likelihood of missed cancer detection for clinical use. In practice, determining the balance between sensitivity and specificity requires further exploration through cost-effectiveness studies in various intended populations.

In our study, we selected metabolites that are commonly dysregulated across three different types of cancer and uniquely dysregulated within specific cancer subtypes. These metabolites were mapped in KEGG metabolic pathways, providing robust biomarkers for our model (34). Specifically, the features across cancer types were selected (LC vs. Non-LC, GC vs. CRC) to build the tumor classification model. Several studies have shown that gastrointestinal cancers shared similar mechanisms (24). Thus, we first compared the LC and gastrointestinal cancers (including GC and CRC), these selected biomarkers were mainly involved in amino acid metabolism. Furthermore, the biomarkers involved in carbohydrate metabolism were chosen to discriminate against GC and CRC. In a meta-analysis aimed at identifying biomarkers distinguishing between GC and CRC, it was observed that galactose metabolism was significantly enriched only in the GC group (24), consistent with our study findings. Additionally, utilizing TCGA pan-cancer database analysis, dysregulation of genes involved in fructose and mannose metabolism was observed specifically in GC patients, whereas no such dysregulation was found in the CRC group (35).

There are still several limitations in our study. The comprehensive follow-up was lacking for all non-cancerous participants to ensure their non-cancerous status, which could potentially result in an inflated false positive rate (FPR) and an underestimated positive predictive value (PPV). Besides, it’s noteworthy that the variability in metabolites levels among both healthy individuals and those with diseases necessitates the validation of metabolic biomarkers using independent assay platforms and external validation cohorts. When millions of individuals undergo cancer screening, even a highly selective assay utilized in population screening may produce a significant number of false positives. Hence, it’s crucial to eliminate false positives, as they could lead to significant and unnecessary stress for the individual. Furthermore, the current study only analyzed three cancer types, although these are among the most prevalent cancers in China, expanding the analysis to include other prevalent and significant cancers in the multi cancer strategy, such as liver, kidney, gynecological, and pancreatic cancers, would certainly broaden its clinical applicability.

## Conclusions

In summary, we have developed a urine-based metabolic biomarker panel for early detection of multi-cancers. The selected metabolic panel, identified through the integration of algorithms and biological significance, effectively differentiated cancer patients from healthy controls and those with benign conditions. Additionally, a cancer classification model accurately predicted tumor origin. Our findings demonstrated that urinary metabolomics can be applied as a universally applicable, straightforward, and cost-effective method for early cancer detection across large populations. In the future, with larger-scale external validation and the expansion to more types of cancer, the pan-cancer screening method based on urine metabolomics has the potential to be translated into clinical settings to support precision medicine, aiding in the identification and localization of common malignancies.

## Data Availability

The raw data supporting the conclusions of this article will be made available by the authors, without undue reservation.
