# Supplementary material for: Development of a urine-based metabolomics approach for multi-cancer screening and tumor origin prediction
Source: Front Immunol. 2024 Dec 13;15:1449103. doi: 10.3389/fimmu.2024.1449103 (PMC11671364; doi:10.3389/fimmu.2024.1449103)
Supplement: Supplementary file 1 [file DataSheet1.docx]

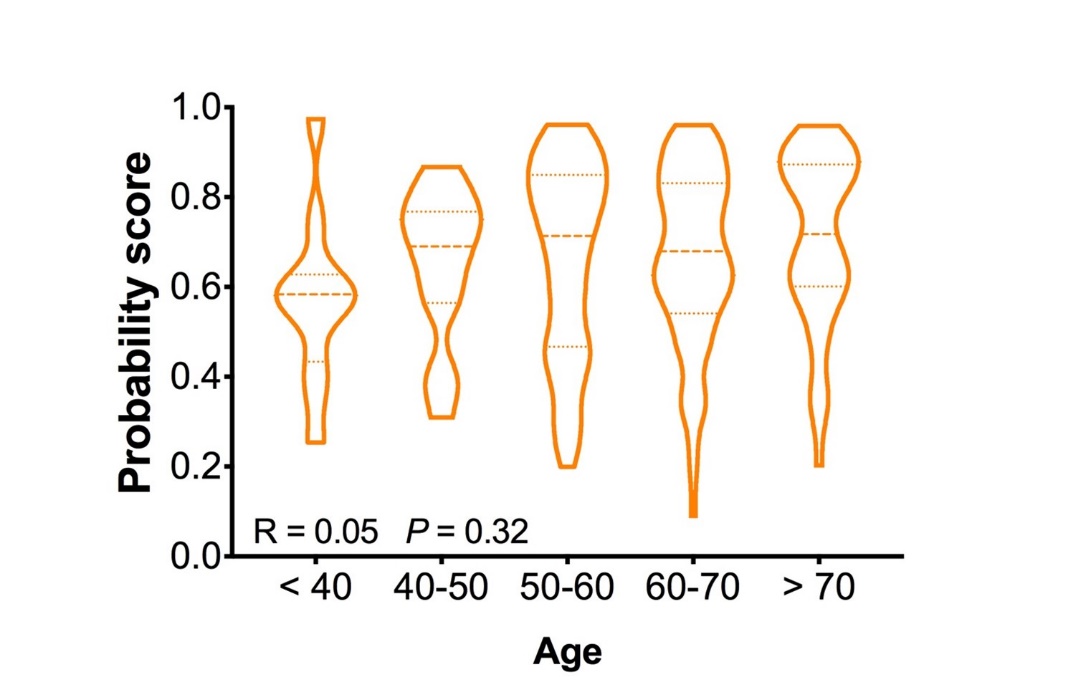


**Figure S1.** The violin plot showing the model probability score distribution of the cancer patients at different age stages in the validation cohort.


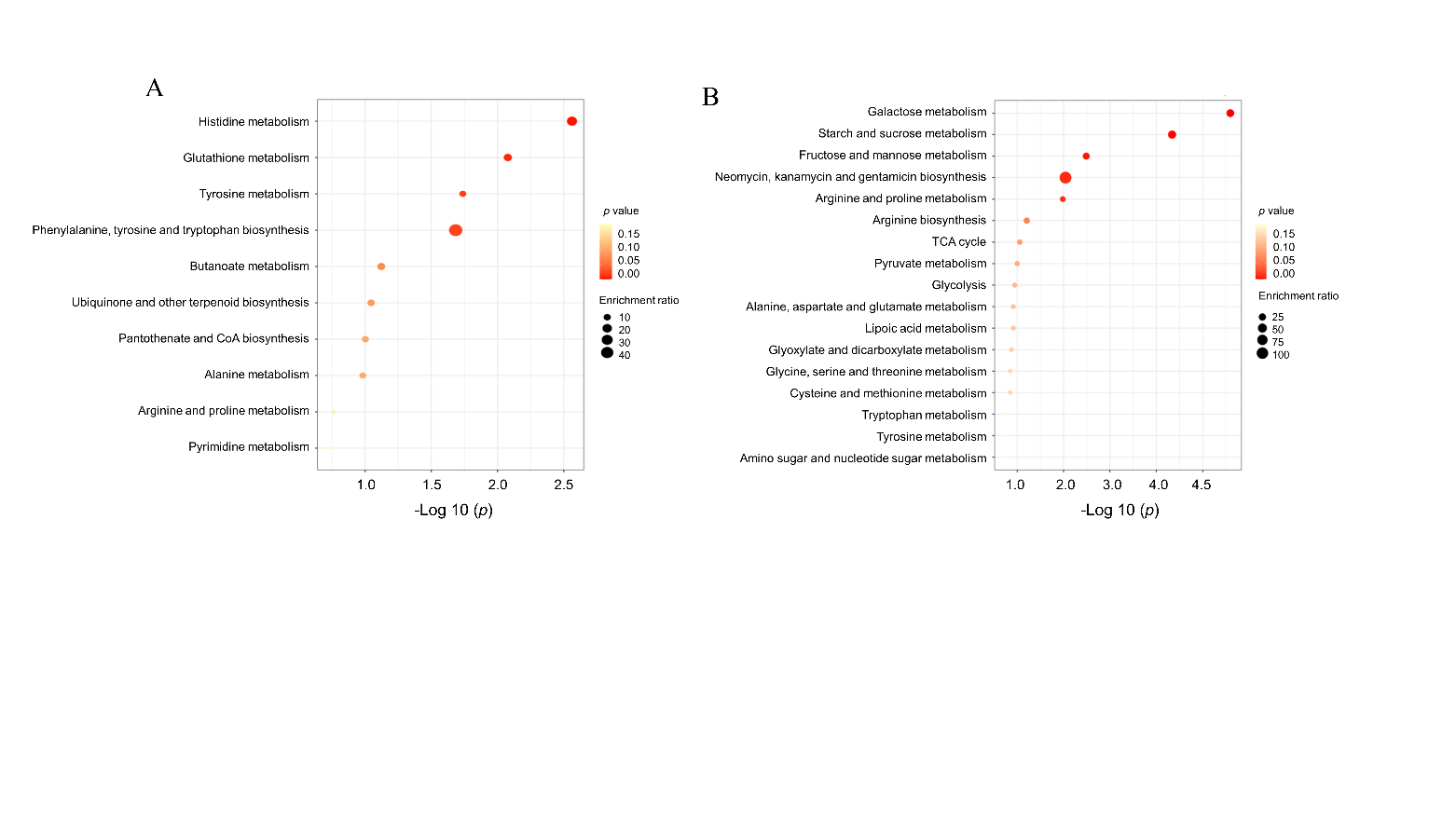


**Figure S2.** Kyoto Encyclopedia of Genes and Genomes (KEGG) metabolic pathways enriched by lasso selected features between lung cancer patients and non-lung cancer groups (A) and between gastric cancer and colorectal cancer groups (B).
